# Supplementary material for: Behavioural Determinants of Appropriate Antibiotic Prescribing for Urinary Tract Infections in Nursing Homes: A Qualitative Study of Stakeholders’ Perspectives
Source: Antibiotics (Basel). 2025 Dec 19;15(1):5. doi: 10.3390/antibiotics15010005 (PMC12837733; doi:10.3390/antibiotics15010005)
Supplement: Supplementary file 1 [file antibiotics-15-00005-s001.zip › Supplementary file S1-Coding tree.docx]

**Supplementary file S1: Coding tree**

Table S1. Coding tree

| **TDF domain** | **Behavioural determinant** | **Stakeholder** |
| --- | --- | --- |
| Knowledge | Lack of knowledge on guidelines (B) | NT |
|  | Lack of knowledge on prescribing practices of TPs (B) | CP |
|  | Lack of knowledge on resident (B) | P |
|  | Lack of monitoring antibiotic use (B) | M |
| Skills | Anamnesis and clinical examination (differential diagnosis) | TP |
|  | Consulting of (inter)national guidelines (F) | TP, P |
|  | Interprofessional communication (F) | TP, P, NT |
|  | Communication with residents and relatives (F) | TP, NT |
|  | Structured documentation of medical record (F) | TP |
|  | Multidisciplinary consultations (F) | TP, P, NT |
|  | Implementing a policy in the NH (F) | CP |
|  | Lack of observation of resident (B) | NT |
|  | Urine sample collection (B) | NT, R |
|  | Ability to swallow medication | R |
|  | Difficulties in communicating symptoms (B) | R |
| Beliefs about capabilities | Self-confidence supported by guidelines or experience (F) | TP,P |
|  | Variable trust in nursing staff | TP, NT, R, M |
|  | Trust in physician - looking up to physician | P, R, NT, M |
|  | Low self-confidence (B) | P, NT |
|  | Low capability of residents to communicate signs and symptoms (B) | NT, R |
| Optimism | Optimism about current situation: decreased antibiotic consumption and good quality of care in NH | TP, P, R, M |
|  | Pessimism about current situation: high antibiotic consumption and resistance | TP, P, NT, M |
|  | Optimism about future AMS-intervention and appropriate antibiotic use (F) | P |
|  | Pessimism about feasibility and effectiveness of a future AMS intervention (B) | P |
| Intention | To support appropriate antibiotic use - to follow guidelines (F) | TP, P, NT |
|  | To reduce unnecessary treatment (F) | TP |
|  | To provide personalised care (F) | P, NT |
|  | To focus on prevention (F) | NT, M |
|  | Resolving the resident's symptoms as quickly as possible (B) | R, NT |
|  | Resistance to medication intake (B) | R |
| Goals | Appropriate antibiotic use (F) | TP, P, M |
|  | Implementing an antibiotic policy in NH (F) | CP |
|  | Avoiding AMR (F) | TP |
|  | Personalised care (F) | P |
|  | Prioritising residents’ comfort | NT, R, M |
| Beliefs about consequences | Discrepancy in perceived risk of AMR in residents | TP, P |
|  | Low risk for severe infections (F) | TP, P |
|  | Positive consequences of appropriate antibiotic use: resident QOL, faster healing process, decrease in side effects of antibiotics, benefits for society (F) | TP, P |
|  | Negative consequences of inappropriate antibiotic use: AMR, societal costs, side effects (F) | TP, P, NT, R |
|  | Low perceived risk of AMR in residents (B) | TP, NT, M |
|  | Benefits of prescribing antibiotics: necessary for the treatment of UTI, relief of symptoms; (B) | NT, R, M |
|  | Negative consequences of not prescribing antibiotics: the longer you wait, the worse it gets (B) | NT, R, M |
| Reinforcement | Low reinforcement for appropriate prescribing (B) | TP |
|  | Positive feedback of colleagues (F) | P |
|  | Authoritarian behaviour physician (B) | P, NT |
|  | Previous personal experience of UTI: very painful | NT, R, M |
|  | Symptoms improved after antibiotics were started | R |
| Emotion | Fear of missing complications (B) | NT, TP, CP, R |
|  | Fear for new medications (B) | R |
|  | Reassured by follow-up HCP (F) | NT, R, M |
|  | Feelings of powerlessness (B) | NT, P, R, M |
|  | Disappointment of non-evidence-based practice | P |
|  | Disappointed in communication of HCPs to relatives (B) | R |
| Memory, attention and decision process | Lack of self-reflection and monitoring (B) | NT, P, TP, CP |
|  | Critical reflection and monitoring are important tools (F) | TP, P, NT, M |
|  | Personalised care | NT, R, M |
|  | (Over)alertness on UTIs (B) | NT |
|  | Increased attention to subject of recent training (F) | NT |
|  | Follow-up: preference to take action immediately versus wait and see (B) | R, NT |
| Behaviour regulation | Medication review and interdisciplinary consultations as reflection tool (F) | TP, P |
|  | Action planning (future): Developing an antibiotic policy in NH (F) | CP, P, NT |
|  | Lack of modalities to implement an antibiotic policy (B) | CP,P |
|  | Preparation emergency medication with first line antibiotics (F) | CP, P |
|  | Screenen new residents for AMR (F) | CP |
|  | Self-monitoring | P |
|  | Consultations with GP and relatives (F) | NT |
|  | Observations | NT |
|  | Action planning (future): Consultation with TP and nurse about prevention of recurrent UTI (F) | R |
| Social/professional role and identity | Responsible for decision making on diagnosis, treatment and follow-up of resident but not accessible (B) | TP |
|  | Interprofessional collaboration (F) | TP, P, NT, M |
|  | Advising role for HCPs (F) | CP |
|  | Developing antibiotic policy in NH (F) | CP, P, M |
|  | Providing education of policy to NH staff and residents and relatives (F) | CP |
|  | Delivery of antibiotics | P |
|  | Prospective audit and feedback - check of appropriateness not feasible (B) | P |
|  | Potential role in retrospective audit and feedback - surveillance (F) | P |
|  | Education of guidelines (F) | P |
|  | Providing practical information on medication use (F) | P |
|  | Observation and signalling - knowledge of resident's baseline | NT, R |
|  | Communication: briefing to colleagues, contacting TPs/pharmacists, informing residents and relatives | NT, TP |
|  | Follow-up of resident (prevention and treatment - safety netting) | NT |
|  | Daily (intimate) care | NT |
|  | Audit and feedback (F) | NT |
|  | Stimulating (over)alertness (B) | R |
|  | Goal priority setting (F) | M |
|  | Proper functioning of electronic medical records (F) | M |
| Social influence | Hierarchical relations with TP being dominant (B) | NT, P, TP, CP, R |
|  | Social pressure to prescribe (B) | NT, R |
|  | Influence of colleague TPs (F) | TP |
|  | Resistance of (older) physicians to external influence on prescribing practices (B) | TP |
|  | Lack of influence (B) | CP, P, NT |
|  | Good interprofessional relations - local pharmacy (F) | P |
|  | Gatekeeper - information source | NT |
|  | Variation in involvement of relatives | R |
|  | Communication of symptoms - difficult in case of cognitive impairment (B) | R |
|  | Informing TPs of NH policy/guidelines through empowering CP or nursing staff (F) | M |
|  | Supporting continuing professional education (F) | M |
| Environmental context and resources | Lack of time and high workload (B) | TP, P, NT |
|  | Working in shifts - lack of continuity (B) | NT |
|  | Limited availability of trimethoprim (first-choice) (B) | TP, P |
|  | Difficulty in contacting TPs, especially during out of office hours (B) | TP |
|  | Fewer different TPs per NH (F) | TP |
|  | Limited mandate CP (B) | CP |
|  | Lack of monitoring antibiotic use (B) | CP, P, M |
|  | Absence of an antibiotic policy (B) | CP, M |
|  | Lack of access to medical information (B) | P |
|  | Lack of integrated interprofessional communication system (B) | P, M |
|  | Lack of support for interdisciplinary consultations (B) | P |
|  | Lack of financial support role in audit and feedback (B) | P, M |
|  | Diagnostic uncertainty, frailty (B) | R |
|  | Lack of guidelines (B) | TP, P |
|  | Lack of support for interprofessional collaboration (B) | P |

TDF = Theoretical Domains Framework, NT = nursing team, P = pharmacist, TP = treating physician, CP = coordinating physician, M = nursing home management, R = residents and relatives, B = barrier, F = facilitator, NH = nursing home, AMR = antimicrobial resistance, AMS = antimicrobial stewardship, QOL = quality of life, HCP = healthcare professional, UTI = urinary tract infection
