# Supplementary material for: Behavioural Determinants of Appropriate Antibiotic Prescribing for Urinary Tract Infections in Nursing Homes: A Qualitative Study of Stakeholders’ Perspectives
Source: Antibiotics (Basel). 2025 Dec 19;15(1):5. doi: 10.3390/antibiotics15010005 (PMC12837733; doi:10.3390/antibiotics15010005)
Supplement: Supplementary file 1 [file antibiotics-15-00005-s001.zip › Supplementary file S2-Topic guides.docx]

**Supplementary file S2: Topic guides**

Healthcare professional interview

Hello, thank you for taking the time today to participate in this interview. I am Indira, doctoral researcher (pharmacist by training) in the team of Prof. Veerle Foulon at KU Leuven. We are conducting a study on the rational use of antibiotics in Flemish nursing homes. With this research, we aim to understand the therapy choices regarding antibiotic use in nursing homes. This includes aspects such as diagnosis, prescribing, duration, possible de-escalation, and the impact on both health outcomes for individual residents and on the well-being of the wider community. The insights gained from this study will help us to strengthen rational antibiotic use in nursing homes. We focus on two stages of the process: the step where a suspected urinary tract infection (UTI) is identified in a resident, and the step where antibiotics are considered. In this interview, we would like to hear about your daily practice. If you agree, the conversation will be audio recorded and later transcribed. All data will be handled confidentially, and anonymity of participants is guaranteed in the reporting.

There are no right or wrong answers, as we are mainly interested in understanding the different perspectives of healthcare professionals on this topic. Please feel free to answer openly.

If you have no questions, we will now begin the interview. To start, we have a few general questions about your daily practice.

**Introductory questions**

- Could you briefly introduce yourself and describe your role in the nursing home?
- How long have you been working here?
- What does your daily work in the nursing home usually look like?
- How do you get in contact with residents, and what is your role in the process of diagnosis and treatment?

**Main topic**
We would now like to focus on the rational use of antibiotics for UTIs in nursing homes.

**Examples of guiding questions**

- When you are contacted about a possible UTI in a resident, how does that process go?
- What are the different steps?
  - How is the situation first noticed?
  - What observations or tests are performed?
  - How do you assess reliability of symptoms and results?
  - What information is taken into account before starting antibiotics?
  - Which factors influence decision-making?

How evidence-based do you consider the use of antibiotics for UTIs in nursing homes to be?

**Background**
In implementation research, an overarching theoretical framework is often used to identify and analyse behavioural determinants. These domains are summarised in the following figures. They include internal domains (personal factors) and external domains (factors related to social influences, the nursing home setting, and the broader context). We will start by zooming in on the internal domains.

Table S2. Topic guide of healthcare professional interview

| **Theme** | **Main question** | **Additional question** |
| --- | --- | --- |
| Knowledge | What do you know about the prevalence, diagnosis, and treatment of UTIs in nursing homes? | Knowledge about UTIs in nursing homes? Knowledge about guidelines? Knowledge about residents? |
| Skills | Which skills do you need to achieve rational antibiotic use? | Which additional skills would you like to acquire? In what way? |
| Belief in capabilities | How does your self-confidence influence the current process of diagnosing and treating UTIs in nursing homes? | What influences this self-confidence? |
| Optimism | How optimistic are you that rational antibiotic use in nursing homes could improve health outcomes for residents and society? | In what way does this optimism influence your actions? |
| Intention | How willing are you to support rational antibiotic use for UTIs according to the guidelines? | What is your attitude towards the guidelines? |
| Goals | How important do you find rational antibiotic use as part of good patient care in nursing homes? | Which standard are you striving to achieve? |
| Beliefs about consequences | What are the benefits and drawbacks you see of optimizing rational antibiotic use in nursing homes? | What is the balance between potential benefits and harms? |
| Emotion | How do you feel in your current practice regarding rational antibiotic use in the treatment of residents with UTIs in nursing homes? | What are your concerns? |
| Memory, attention and decision processes | What are the different steps you take in your decision-making process? | Which factors influence this decision-making process? |
| Behavioural regulation | How is your individual practice or temporary practice within the nursing home different from what it should ideally be? | Which of these different steps are you willing to carry out? |
| Reinforcement | What are the benefits and rewards of optimizing the process of diagnosing and treating UTIs in nursing homes? | Which stimulus motivates you? |
| Social/professional role and identity | What is your current role and that of other caregivers in diagnosing and treating UTIs in nursing homes? | Which potential roles do you or other caregivers see? |
| Social influence | How do other people influence your current practice regarding rational antibiotic use in nursing homes? | What is the influence of family members or other caregivers? |
| Environmental context and resources | What is the impact of the specific context of the nursing home on antibiotic use? | What is the impact of access to resources on rational antibiotic use? |

Resident and/or relative interview

Hello, thank you for making time today for this interview. My name is Indira, I am a researcher at KU Leuven. I am here today, in the context of the research I am conducting, to have a conversation with you about antibiotics. I estimate this will take about half an hour.

To begin, may I ask if it is okay for me to record this interview? This will allow me to process your answers later on, of course respecting your privacy. I have brought an information form for you with all the necessary details about our study. We will go through it together – review the information, check if everything is clear, provide my contact details in case you have questions, and finally ask you to sign the informed consent form.

Before we get started, I would like to emphasize that there are no right or wrong answers.

I am conducting research on the appropriate use of antibiotics in nursing homes. By this we mean treatment according to scientific guidelines: only when necessary, with the right choice of antibiotic, the right dosage, the right timing between doses, and the correct duration. The aim is, on the one hand, to help the person with an infection recover as well as possible and, on the other hand, to minimize health risks for society. Our focus is on the treatment of urinary tract infections.

The goal of this interview is to learn about your perspective on the use of antibiotics, as a resident of a nursing home.

To get to know each other a little: could you briefly introduce yourself?

- How old are you?
- How long have you been living in the nursing home?

Your care providers indicated that we could interview you because you recently received antibiotics for a urinary tract infection. Could you tell me a bit more about that?

- What symptoms did you have?
- What type of infection was it?
- How was the infection diagnosed?
- How often do you experience such infections?
- Was this also the case before you moved into the nursing home? How was it handled then?
- Which antibiotics did you use at that time?
- Which healthcare providers were involved in your treatment?

**EMOTIONS**

- What did you think about the way your infection was treated?
- How was it for you to have to take antibiotics?

**ATTITUDE**

- Some people are concerned because antibiotics are used a lot. What is your opinion about antibiotics?
- There are many public campaigns about antibiotics, for example that antibiotics do not work against a common cold. What impact do these campaigns have on you?

**KNOWLEDGE**

- What do you know about the advantages and disadvantages of antibiotics?
- There is often talk about resistant bacteria. What does that mean to you?

**EXPECTATIONS**

- Which healthcare providers would you prefer to see if you have an infection?
- With whom would you like to discuss your antibiotic treatment?
- What would you like to know from them?

That was everything I wanted to ask you. Thank you very much for your answers. Before we close this interview, is there anything important regarding the use of antibiotics that has come to your mind and that we have not yet discussed?

If this interview has raised specific questions about your own antibiotic treatment, you can always contact the staff of the nursing home or your general practitioner.
