# Supplementary material for: Behavioural Determinants of Appropriate Antibiotic Prescribing for Urinary Tract Infections in Nursing Homes: A Qualitative Study of Stakeholders’ Perspectives
Source: Antibiotics (Basel). 2025 Dec 19;15(1):5. doi: 10.3390/antibiotics15010005 (PMC12837733; doi:10.3390/antibiotics15010005)
Supplement: Supplementary file 1 [file antibiotics-15-00005-s001.zip › Supplementary file S3-COREQ checklist.docx]

**Supplementary file S3: Consolidated criteria for reporting qualitative studies (COREQ) checklist**

Table S3: COREQ checklist

| **No** | **Item** | **Guide questions/description** | **Response** |
| --- | --- | --- | --- |
| **Domain 1: Research team and reflexivity** | | |  |
| Personal Characteristics | | |  |
| 1. | Interviewer/facilitator | Which author/s conducted the interview or focus group? | We mentioned this in the Methods. (Data collection section) |
| 2. | Credentials | What were the researcher's credentials? *E.g. PhD, MD* | We mentioned this in the Methods. (Study design section) |
| 3. | Occupation | What was their occupation at the time of the study? | We mentioned the researchers’ affiliations after the authors’ names. |
| 4. | Gender | Was the researcher male or female? | We mentioned the researchers’ gender in the Methods. (Study design section) |
| 5. | Experience and training | What experience or training did the researcher have? | We mentioned the researchers’ experience in the Methods. (Study design section) |
| Relationship with participants | | |  |
| 6. | Relationship established | Was a relationship established prior to study commencement? | Yes, we mentioned in the Methods (Participants section) that the call was also launched in the researchers’ professional networks. |
| 7. | Participant knowledge of the interviewer | What did the participants know about the researcher? e*.g. personal goals, reasons for doing the research* | At the beginning of each interview, we explained the reasons for doing the research and the professional backgrounds of the researchers. This is mentioned in the topic guides we included as Supplementary data. |
| 8. | Interviewer characteristics | What characteristics were reported about the interviewer/facilitator? e.g. *Bias, assumptions, reasons and interests in the research topic* | We mentioned the researchers’ motivation to conduct this study in the Methods. (Study design section) |
| **Domain 2: study design** | | |  |
| Theoretical framework | | |  |
| 9. | Methodological orientation and Theory | What methodological orientation was stated to underpin the study? *e.g. grounded theory, discourse analysis, ethnography, phenomenology, content analysis* | We described the use of the Theoretical Domains Framework (TDF) to develop the topic guide in the Study design section and to guide the data analysis in the Data analysis section. |
| Participant selection | | |  |
| 10. | Sampling | How were participants selected? *e.g. purposive, convenience, consecutive, snowball* | We described in the Participants section that NHs were selected using purposive sampling. |
| 11. | Method of approach | How were participants approached? e*.g. face-to-face, telephone, mail, email* | We mentioned in the Methods (Participants section) that the study invitation was initially sent by mail to four NHs that met the inclusion criteria. |
| 12. | Sample size | How many participants were in the study? | In the Results (Participants’ characteristics), we reported that a total of 63 individuals participated in the interviews and focus groups. |
| 13. | Non-participation | How many people refused to participate or dropped out? Reasons? | We described in the Results (General characteristics and antibiotic policy of NHs) that thirteen NHs were approached and nine NHs declined participation due to time constraints. |
| Setting | | |  |
| 14. | Setting of data collection | Where was the data collected? e*.g. home, clinic, workplace* | In the Methods section (Data collection), we reported that data were collected through a combination of in-person interviews in the NHs and online interviews conducted via Microsoft Teams. |
| 15. | Presence of non-participants | Was anyone else present besides the participants and researchers? | In the *Data collection* section, it is noted that Master’s students in Pharmaceutical Care acted as observers. |
| 16. | Description of sample | What are the important characteristics of the sample? *e.g. demographic data, date* | An overview of the participants’ characteristics is presented in Table 2. |
| Data collection | | |  |
| 17. | Interview guide | Were questions, prompts, guides provided by the authors? Was it pilot tested? | Yes, we mentioned in the Methods that a topic guide was developed and refined after pilot testing. |
| 18. | Repeat interviews | Were repeat interviews carried out? If yes, how many? | NA |
| 19. | Audio/visual recording | Did the research use audio or visual recording to collect the data? | Yes, we reported in the Methods (Data collection section) that we used audio recordings. |
| 20. | Field notes | Were field notes made during and/or after the interview or focus group? | Yes, we mentioned in the Data collection section that we made field notes after the interviews. |
| 21. | Duration | What was the duration of the interviews or focus group? | The duration of the interviews (30-60 min) and focus groups (90 min) is reported in the Results section (Participants’ characteristics). |
| 22. | Data saturation | Was data saturation discussed? | We reported in the Methods (Participants section) that participants were recruited until data sufficiency was reached. |
| 23. | Transcripts returned | Were transcripts returned to participants for comment and/or correction? | No |
| **Domain 3: analysis and findings** | | | |
| Data analysis | | | |
| 24. | Number of data coders | How many data coders coded the data? | We reported in the Data analysis section that the data were coded by three researchers. (IC, SL and KC) |
| 25. | Description of the coding tree | Did authors provide a description of the coding tree? | Yes, the coding tree is described in the Results section, with reference to the Supplementary Data where the full coding tree is provided. |
| 26. | Derivation of themes | Were themes identified in advance or derived from the data? | In the data analysis, we reported using a combined approach: a deductive method to define the themes (i.e. TDF domains) and an inductive method to identify the subthemes (i.e. behavioural determinants). |
| 27. | Software | What software, if applicable, was used to manage the data? | We mentioned that our data analysis was supported by NVivo 14 Software. |
| 28. | Participant checking | Did participants provide feedback on the findings? | In the data collection section, we described that Master’s students were present as observers and shared their main observations afterwards, allowing participants to provide feedback. |
| **Reporting** | | | |
| 29. | Quotations presented | Were participant quotations presented to illustrate the themes / findings? Was each quotation identified? e*.g. participant number* | Yes, in Table 3 each key behavioural determinant is accompanied by an illustrative quote, together with the pseudonym of the participant, as detailed in the Data Collection section. |
| 30. | Data and findings consistent | Was there consistency between the data presented and the findings? | Yes, illustrative quotes supporting each key behavioural determinant are provided in Table 3, ensuring a clear link between participants’ accounts and the reported results. |
| 31. | Clarity of major themes | Were major themes clearly presented in the findings? | Yes, the major themes and subthemes are presented in Table 3 and Figure 1, which provide the basis for the explanation of the results in the Results section. |
| 32. | Clarity of minor themes | Is there a description of diverse cases or discussion of minor themes? | Yes, as some subthemes were connected to multiple TDF domains, we illustratively described the professional roles undertaken by the different stakeholders and beliefs about consequences. These two TDF domains were not retained as primary themes. |
